# Differential Expression of Rubisco in Sporophytes and Gametophytes of Some Marine Macroalgae

**DOI:** 10.1371/journal.pone.0016351

**Published:** 2011-01-20

**Authors:** Chao Wang, Xiaolei Fan, Guangce Wang, Jianfeng Niu, Baicheng Zhou

**Affiliations:** 1 Key Laboratory of Experimental Marine Biology, Institute of Oceanology, Chinese Academy of Sciences, Qingdao, China; 2 Graduate University of the Chinese Academy of Sciences, Beijing, China; 3 Qingdao Institute of Bioenergy and Bioprocess Technology, Chinese Academy of Sciences, Qingdao, China; University of Canterbury, New Zealand

## Abstract

Rubisco (ribulose-1, 5-bisphosphate carboxylase/oxygenase), a key enzyme of photosynthetic CO_2_ fixation, is one of the most abundant proteins in both higher plants and algae. In this study, the differential expression of Rubisco in sporophytes and gametophytes of four seaweed species — *Porphyra yezoensis*, *P. haitanensis*, *Bangia fuscopurpurea* (Rhodophyte) and *Laminaria japonica* (Phaeophyceae) — was studied in terms of the levels of transcription, translation and enzyme activity. Results indicated that both the Rubisco content and the initial carboxylase activity were notably higher in algal gametophytes than in the sporophytes, which suggested that the Rubisco content and the initial carboxylase activity were related to the ploidy of the generations of the four algal species.

## Introduction

Algae are an ancient group of unicellular and multicellular organisms. Unlike higher plants, algae have a life history style that is diverse and displays three different styles: species in which the gametophyte predominates, represented by monoecious *Porphyra yezoensis* (Rhodophyte) and dioecious *P. haitanensis* (Rhodophyte); species in which the gametophyte and the sporophyte are of equal dominance, represented by *Bangia fuscopurpurea* (Rhodophyte); and species in which the sporophyte is predominant, represented by *Laminaria japonica* (Phaeophyceae). In contrast to higher plants, both the gametophytes and the sporophytes of algae can photosynthesize and survive independently as autotrophic organisms. Algae are thus ideal organisms for comparative studies on the life histories of different generations of photosynthetic organisms as well as for studies on photosynthesis.

Ribulose-1, 5-biphosphate carboxylase-oxygenase (Rubisco) — a key enzyme of carbon assimilation that is widely distributed in photosynthetic organisms [1] — is crucial for comparative studies among generations of algae. It is also one of the most abundant proteins, accounting for more than 50% of the total soluble protein in C3 plants [2]. In both higher plants and green algae the large subunit (LSU) and small subunit (SSU) of Rubisco are respectively encoded by the chloroplast genome and the nuclear genome [3]. In Rhodophyta and Phaeophyta, however, both the LSU and the SSU are encoded by the chloroplast genome in an operon [4], so the LSU, which provides all the catalytically-essential residues, can directly reflect the activity of the mature Rubisco [5]. A comparative study on Rubisco in different organisms could therefore provide interesting insights into disparities in generations of algae.

In higher plants the abundance of both Rubisco and carboxylase activity are quite different among organs and during the development of leaves and seeds [6]–[8], while in algae, the activity rates of Rubisco are reported to be conspicuously different among the stipe, meristem and blade of *Laminaria setchellii* [9]. The possible function of Rubisco during the development of organisms has not, however, been investigated. On most occasions, the function of Rubisco has been attributed to the assumed different carbon fixation efficiency. In recent years, it was reported that Rubisco acted in a previously-undescribed metabolic context without the Calvin cycle to increase the efficiency of carbon use during the formation of oil in developing embryos of *Brassica napus* L. (oilseed rape) [10]. Comparative studies on Rubisco activity during algal life cycles, could improve understanding of its function in seaweeds. In this study, we took four species of algae — *Porphyra yezoensis*, *P. haitanensis*, *B. fuscopurpurea* (Rhodophyte) and *L. japonica* (Phaeophyceae), which all have dimorphic life cycles, consisting of haploid gametophytes and diploid sporophytes — as typical examples for three types of life history. Comparative research on the levels of mRNA, transcription, enzyme activity and protein expression was then undertaken to investigate differences in Rubisco activity in gametophytes and sporophytes during the process of carbon assimilation.

## Materials and Methods

### Materials

Filamentous sporophytes of *Porphyra* were cultivated in a 12 hr photoperiod of 36 µmol·m^−2^·s^−1^ fluorescent illumination at 17°C with constant air bubbling. The freshly collected leafy gametophytes were preserved at −20°C after drying in a shady breezy place. The dried blades were resuscitated in 4°C filtrated culture for about a week before use, after which healthy mature blades were selected.

The sporophyte of *L. japonica* was freshly collected and cultivated in a 12 hr photoperiod of 80 µmol·m^−2^·s^−1^ fluorescent illumination at 9°C for about a month. The gametophytes were cultivated in a 12 hr photoperiod of 20 µmol·m^−2^·s^−1^ fluorescent illumination at 9°C.

Sporophytes and gametophytes of *B. fuscopurpurea* were both kept in a laboratory culture system. The sporophyte was cultivated in a 12 hr photoperiod of 24 µmol·m^−2^·s^−1^ fluorescent illumination at 18°C and the gametophyte cultivated in the same photoperiod at 40 µmol·m^−2^·s^−1^ fluorscent illumination at 13°C.

All the algal culture media was renewed on a weekly basis with bacterial-free seawater containing 0.1 mM KH_2_PO_4_ and 0.1 mM NaNO_3_. Before use, the algal material was cleaned three times with pre-cooled filtrated seawater and again (for another three times) with pre-cooled distilled water. Water remaining on materials was wiped away at 4°C in dark conditions. After being weighed, algal material was preserved in liquid nitrogen for later use.

### Chloroplast genomic DNA (ctDNA) isolation from leafy gametophytes of *P. yezoensis*

One gram of material was homogenized at 0°C in 10 mL buffer I (containing 5 mM EDTA, 1 mM MgCl_2_, 1 mM MnCl_2_, 2 mM NaNO_3_, 0.5 mM K_2_HPO_4_ and 0.1 M sucrose). After centrifuging at 350 g at 4°C for 5 min, the supernatant was transferred to another tube and centrifuged at 2,000 g at 4°C for 10 min. The supernatant was discarded and the nuclei pellets were collected. Buffer II (50 mM Tris, 25 mM EDTA, 2% SDS, 50 µg/mL Proteinase K, pH 8.0) was added to resuspend the pellets followed by incubation at 40°C for 3 hr. The pellets were discarded after centrifugation at 12,000 g at 4°C for 10 min. One volume of saturated phenol, saturated phenol-chloroform-isoamyl alcohol (25∶24∶1) and chloroform-isoamyl alcohol (24∶1) were then added, sequentially, to the sample. The aqueous phase was collected and two volumes of absolute ethanol with 0.1 volume (approximately 50 mL) sodium acetate (3 M pH 5.2) were added and gently mixed. The sample was left for at least 20 min at 20°C and then centrifuged at 12,000 g at 4°C for 30 min, after which the supernatant was discarded. After washing with 70% ethanol and drying at room temperature, the pellets were dissolved in 10–50 mL of pure water and preserved at −20°C for use.

### Probe synthesis and its hybridizing efficiency determination

The *rbcL* (gene encoding large subunit of Rubisco) of *P. yezoensis* was amplified in a 25 µL PCR reaction system containing 2.5 µL 10× Reaction Buffer (with 15 mM MgCl_2_), 2.5 µL of dNTPs (2 mM each), 0.5 µL of each primer (10 µM) including rbcl 1 (5′- CTGCAGAAATGGGTTACTGGGATG - 3′), rbcl 2 (5′- CTCGAGGTCTCAACGAAATCAGCT - 3′), 0.3 U of Taq polymerase (Promega), approximately 5–10 ng of template DNA and a corresponding volume of PCR water. The mixtures were subjected to the following conditions: 95°C for 5 min, followed by 35 cycles of 94°C for 45 s, 45°C for 40 s and 72°C for 80 s, and a final extension at 72°C for 10 min. All reactions were conducted on a Mastercycler gradient (Eppendorf). The products were purified for probe synthesis.

Labeling the DNA with Random primer and determination of the hybridizing efficiency were performed according to the protocol of the Dig High Prime DNA Labeling and Detection Starter Kit I (Roche).

### Northern blot assay

The sporophytic and gametophytic materials of *P. yezoensis* were separately homogenized in liquid nitrogen; 1 mL Trizol reagent (Invitrogen) was added to 100 mg (F_w_) materials and the total RNA was extracted according to the protocol. Northern blot assay was performed according to the protocol of the Dig High Prime DNA Labeling and Detection Starter Kit I (Roche). Prehybridization temperature and hybridization temperature were 50°C, and the incubation temperature with high stringency buffer was 65°C.

### Determination of rbcL gene expression in gametophytes and sporophytes by qPCR

Total RNA of four algae, including gametophytes and sporophytes, were extracted with an RNAprep pure plant kit (Tiangen). Reverse transcription of each DNase-treated RNA template was carried out with random primer and M-MLV Reverse Transcriptase (Promega).

The qPCR was performed on a Bio-Rad iQ5 Multicolor Real-time PCR Detection system (Bio-Rad, Hercules, CA, USA) with the total volume of 25 µL reaction mix containing 12.5 µL of 2× SYBR® Premix Ex Taq™ (TaKaRa Biotechnology Co., Ltd.), 2.5 µL of each primer (2 µM), 2.5 µL of diluted cDNA mix and 5 µL of RNase-free water. Thermal cycling conditions were as follows: an initial temperature of 95°C for 3 min, followed by 35 cycles of 95°C for 15 s, annealing for 40 s, and 72°C for 30 s. The confirmation of the specificity of the PCR product was made by analyzing the dissociation curve at the end of each PCR reaction. To maintain consistency, the baseline was set automatically by the software [11]. Besides, the sequences, amplification efficiencies and corresponding annealing temperatures of all the involved primers for each alga were listed in Table 1. To maximize accuracy, both relative and absolute quantification were carried out to determine the different expression level of *rbcL* in gametophytes and sprorophytes in the four algae.

**Table 1:** Primers used in the qPCR assay.

Algae	Gene		Primer sequences (5′-3′)	Size (bp)	Tem (°C)	En (%)
*PY*	*18S*	F	CGACCGTTTACTGTGAAG	175	58	96.4
		R	GACAATGAAATACGAATGCC			
	*rbcL*	F	GATGTAGTTCTCAGTTTGGTGGTG	168	58	95.6
		R	ACAAGTTTTAGCAGCGTCCCTC			
	*GAPDH*	F	CCAACAAGTGGGAGTAAGCG	104	58	95.7
		R	GGACAGAACCGAACAGCGTA			
*PH*	*18S*	F	CCGTTACTCCTGTGGACCTG	100	56	103
		R	AGGCGAACCTTCAGAGACTTT			
	*rbcL*	F	AACCATTTATGCGTTGGAGAG	291	56	104
		R	GATTTTTTTGACGAGAGTAAGTAGA			
	*GAPDH*	F	GGTGCGTCCAAGCATCTGA	113	56	97.3
		R	TGGGTGTAGTCCTGGTCGTTC			
*BF*	*18S*	F	ACAGGACTTGGGCTCTATTTTG	135	56	100.1
		R	AGATGCTTTCGCAGTGGTTCG			
	*rbcL*	F	AGCCATTTATGCGTTGGAGAG	234	56	100.3
		R	CATTTTTACGAGCCCAGATTGC			
	*ACTIN*	F	CCATCTATGAAGGCTACTCGC	193	56	101.4
		R	GCCATCTCCTTGTCGTAATCC			
*LJ*	*18S*	F	GCCTGAGAAACGGCTACCAC	346	58	105
		R	GACAACCTAATGCCAGCGACAC			
	*rbcL*	F	CTATTGGTCACCCTGATGGTATTC	174	58	100
		R	CTTTCCATAAATCTAACGCTGCTT			
	*ACTIN*	F	CCCATCTACGAGGGTTACGCT	249	58	103.8
		R	GTTTCCGTCGGGGAGTTC			

*PY*: *P. Yezoensis*; *PH*: *P. Haitanensis*; *BF*: *B. Fuscopurpurea*; *LJ*: *L. Japonica*; F: Forward primer; R: Reverse primer; E_n_, the primer's amplification efficiency.

For relative quantification, two house-keeping genes were used as calibrators for each alga in this study to verify successful reverse transcription and to calibrate the cDNA template. The calibrator genes involved in this study were *18S* (gene encoding small subunit ribosomal RNA), *GAPDH* (gene encoding glyceraldehyde-3-phosphate dehydrogenase) and *ACTIN* (gene encoding β-actin), in which *18S* and *GAPDH* were chosen for *P. yezoensis* and *P. haitanensis*, and the *18S* and *ACTIN* were chosen for *B. fuscopurpurea* and *L. japonica*. Three independent reactions together with negative control of each gene were performed and data analysis was carried out using the 2^−△△Ct^ method [12].

For absolute quantification, standard curve was generated by plotfting the Ct value against the logarithm of the quantity (copy numbers), and three independent serial dilutions of cDNA standard samples were performed at the same time together with unknown samples and negative control reactions [13]. The sample's concentration (ng/µL) was determined by using Qubit fluorometer (invitrogen™). The calculation of the quantities of standard unknown samples and the determination of amplification efficiency were performed as follows:

Molecular weight of standard sample (g/mol): 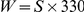
S: base pairs size of single-stranded DNA; 330: the mean molecular weight of single-stranded DNA (Da/bp)

Quantity of standard sample (copies/µL): 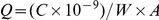
C: sample's concentration; A: Avogadro's constant

Linear equation corresponding to standard curve: 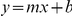



Quantity of unknown sample (copies/µL):
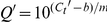
C_t_': the C_t_ value of unknown sample

Amplification efficiency (%):
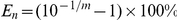



### Preparation of the total protein and determination of total protein content

Algal material was ground into powder in liquid nitrogen. Buffer III (0.1 M Tris, 1 mM EDTA, 50 M Mascorbic acid., 8 M Urea, 0.1 mM PMSF, 1%Triton X-100, 1% β-mercaptoethano, pH 7.8) was added to the powder material (1 mL/g·F_w_) followed by centrifugation at 15,000 g for 30 min. The supernatant was collected as the total protein solution and the total protein content was determined according to the method of Bradford (1976) [14].

### Determination of the chlorophyll a content

N, N-dimethylformamide (DMF) was added into the powder obtained from 1 g algal material to extract chlorophyll a in dark conditions at 4°C for 48 hr. Subsequently, centrifugation was carried out at the maximum rotation speed for 10 min and the supernatant was collected and diluted to a final volume of 25 mL. Chlorophyll a content was calculated according to the formulae of Jeffrey & Humphrey (1975) [15].

*For Phaeophyta:*

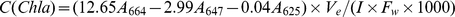



*For Rhodophyta:*

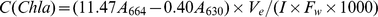
C (Chla): the content of Chlorophyll a (mg/g·F_w_); A: the value of absorbance; V_e_: the total volume of the extraction (mL); I: the diameter of the reaction cuvette (cm); F_w_: the fresh weight of the algal material (g).

### SDS-PAGE electrophoresis

SDS-PAGE was carried out to separate protein components according to Laemmli (1970) [16], using 5% condensing gel and 12% separation gel, and using Tris-Gly buffer (0.125 M Tris, 0.96 M Glycine, 0.5%SDS, pH 8.3) as the electrophoresis buffer. One volume of 2× loading buffer (0.25 M Tris-HCl, pH 6.8, 10% SDS (w/v), 0.1% Bromophenol blue (w/v), 0.5% β-Mercatoethanol (v/v)) was added to the total protein solution and heated in boiling water for 5 min, followed by centrifugation at 13,000 g at 4°C for 5 min. Electrophoresis was carried out at a constant voltage of 80 V for 30 min and then 160 V for 90 min. Gels were stained overnight by shaking in the staining solution containing 0.1% Coomassie brilliant Blue R250, 40% Methanol and 1% Acetic acid for protein visualization.

### Western blot assay for the LSU of Rubisco of *P. yezoensis*

The Rubisco LSU of *P. yezoensis* was determined by separation on 12% SDS-PAGE followed by electrophoretically transferring to PVDF membrane [17]. After rinsing the membrane with 0.01 M phosphate buffered saline (PBS) three times, for 5 min each, incubation with a 1% (w/v) solution of filtered nonfat dried milk (NFDM) in PBS was carried out for 2 hr with shaking at room temperature. The specific polyclonal antibody against the LSU of Rubisco was diluted with the appropriate ratio of 0.01 M PBS and the incubation occurred during a 12 hr period with shaking at 4°C. The negative control underwent the same treatment at the same time using 1% (w/v) Albumin Bovine V (BSA) instead of the first antibody. Between steps, the membrane was washed three times for 15 min with 0.01 M PBS. The cross-reaction between antibody and protein was detected via a chromogenic reaction in which the anti-rabbit secondary antibody conjugated with horse radish peroxidase (Tiangen).

### Determination of Rubisco initial carboxylase activity

Determination of Rubisco initial carboxylase activity was undertaken using the method by Gerard *et al*. (1996) [18].The materials were ground to a fine powder in liquid nitrogen and homogenized in the pre-cooled Rubisco extraction solution at pH 7.6 (1 mL/g·F_w_) containing 40 mM Tris-HCl buffer with 10 mM MgCl_2_, 0.25 mM EDTA and 5 mM reduced glutathione. The homogenate was centrifuged at 2,000 g for 2 min at 4°C, and the supernatant was collected as the crude solution of Rubisco for the measurement of the enzyme activity. The reaction system was prepared as outlined in Table 2. Distilled water was used as the blank and the OD value at 340 nm of the mixture was recorded as the zero-value. The reaction was initiated by adding 0.1 mL of ribulose-1, 5-bisphosphate (RuBP) into the reaction cuvette and the OD values at 340 nm were recorded every 20 seconds for 3 min. The reduction of the OD value in the first minute was used to calculate the initial carboxylase activity. Since the existence of phosphoglycerate in the extraction solution might affect the determination of Rubisco activity, a control without RuBP was needed. The control system was the same as that of the reaction system except that the crude extraction solution was added at the last step. OD values were determined as described above.The following formula was used to calculate Rubisco initial carboxylase activity:

**Table 2:** The reaction system for determining Rubisco initial carboxylase activity.

Reagents	Volume (mL)
5 mM NADH solution	0.2
50 mM ATP solution	0.2
Rubisco extraction solution	0.1
50 mM Creatine Phosphate solution	0.2
0.2 mM NaHCO3 solution	0.2
Reaction buffer*	1.4
160 U/mL Creatinephosphokinase	0.1
160 U/mL Phosphoglycerate kinase	0.1
160 U/mL glyceraldehyde-3-phosphate Dehydrogease	0.1
dd-water	0.3

Reaction buffer* was 0.1 M Tris-HCl buffer (pH 7.8) containing 12 mM MgCl_2_ and 0.4 mM EDTA.



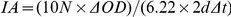
IA: the initial carboxylase activity of Rubisco (mmol CO_2_/mL·min); ΔOD: the margin of the OD value change in the first minute (OD change of the control was detracted); 6.22: the light density per mmol NADH at 340 nm; N: the dilution factor; d: the diameter of the reaction cuvette (cm); Δt: the time (1 min).

### Determination of photosynthetic rate

The experiments of the in vivo chlorophyll fluorescence of PS II (Photosystem II) determination were carried out on Imaging-PAM (Heinz Walz, Effeltrich, Germany), using the automated induction and recovery curve routine provided by the ImagingPam software, with the repetitive application of saturation pulses for assessment of fluorescence. The minimal fluorescence yield (F_0_) was determined using 15 min dark-adapted samples. Saturating pulses were applied to obtain the maximum fluorescence yield (F_m_).

The following formulas were used to calculate the effective PS II quantum yield (Y II) and the optimum quantum yield (F_v_/F_m_), which reflect the actual photosynthetic rate and the potential photosynthetic rate respectively [19]:
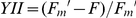


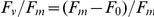



F_m_': the maximum fluorescence yield in illuminated samples

## Results

### Northern blot assay of *rbcL* mRNA of *P. yezoensis*

In order to obtain the DNA fragment coding for the large subunit of Rubisco, the amplification of *rbcL* from the ctDNA of *P. yezoensis* was carried out. The specificity of the amplified fragment was confirmed by sequence analysis of the PCR products and blasting in the NCBI database. The sequencing result showed 99% similarity with the *rbcL* of *P. yezoensis*. Therefore, it was identified as part gene of *rbcL*, which can be used as the probe (RBCL probe) for northern blot assays.

**Figure 1:**
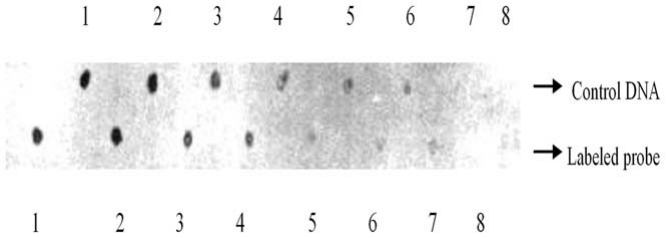
Detection of hybridizing efficiency of the *rbcL* probe from *P. yezoensis*. The Dig High Prime DNA Labeling and Detection Starter Kit I (Roche) was used to detect the hybridizing efficiency of prepared RBCL probe. The probe was labeled with random primer. The control DNA (the first line) which was supplied by the kit was diluted as follows: 1, 1 ng/µL; 2, 10 pg/µL; 3, 3 pg/µL; 4, 1 pg/µL; 5, 0.3 pg/µL; 6, 0.1 pg/µL; 7, 0.03 pg/µL; 8, 0.01 pg/µL; 9, 0 pg/µL. Labeled probe (the second line) was diluted as follows: 1, 1×10^−2^ dilution; 2, 1×10^−4^ dilution; 3, 3.3×10^−4^ dilution; 4, 1×10^−5^ dilution; 5, 3.3×10^−5^ dilution; 6, 1×10^−6^ dilution; 7, 3.3×10^−6^ dilution; 8, 1×10^−7^ dilution; 9, negative control (dd-water).

After labeling, the RBCL probe concentration was estimated to be 100 ng/µL and even a 0.1 pg template produced an obvious hybridizing signal (Fig. 1), indicating that the efficiency which we obtained from the probe was extremely high and sufficient to detect low-abundance mRNAs.

**Figure 2:**
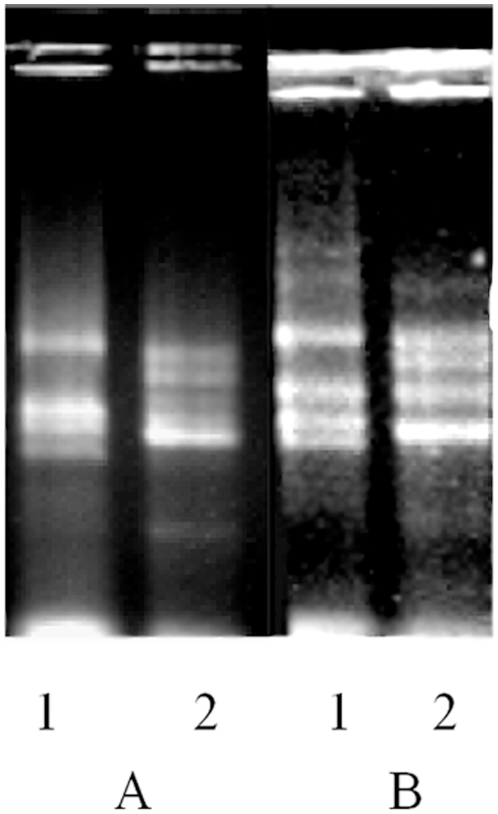
Agarose gel electrophoresis of total RNA of gametophyte and sporophyte of *P. yezoensis*. A, result of native gel electrophoresis. B, result of formaldehyde-agarose gel electrophoresis. 1, Gametophyte. 2, Sporophyte.

**Figure 3:**
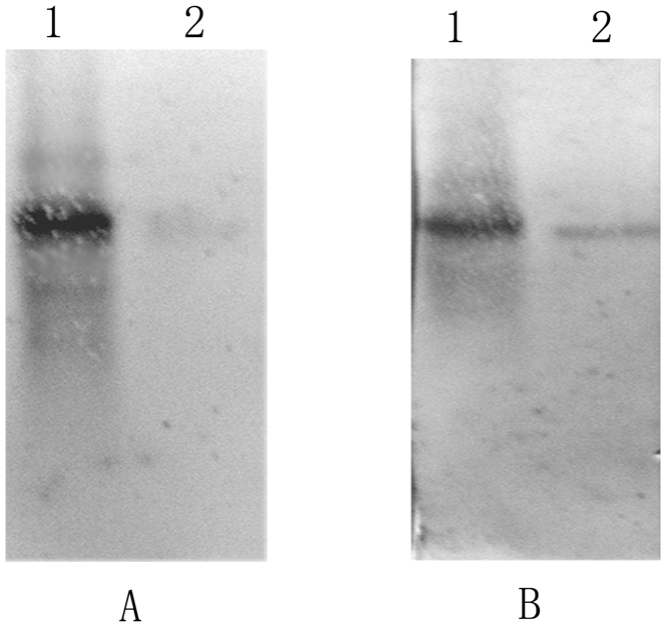
Northern blot results of *P. yezoensis rbcL*. A, comparison result with loading 10 µg total RNA for every lane. B, comparison result with loading total RNA from 25 mg fresh weight material each lane. 1, Gametophyte. 2, Sporophyte.

Figure 2 indicates the total RNA from the gametophyte and sporophyte of *P. yezoensis*. It was obvious that whether we used native gel electrophoresis (Fig. 2-A) or formaldehyde-agarose gel electrophoresis (Fig. 2-B), four major bands were visible, which could be the 28S, 26S, 18S and 13S rRNA, top-to-bottom. In Northern blot analysis of total RNA derived from *P. yezoensis*, a single transcript was detected using the RBCL probe. Results are shown in Figure 3, which indicates that, whether based on fresh weight (Fig. 3-B) or the RNA content (Fig. 3-A), the band of Lane 1 (the gametophyte) was brighter than that of Lane 2 (the sporophyte), suggesting that mRNA of *rbcL* was much more abundant in the gametophyte than in the sporophyte.

### Analysis of rbcL gene expression in gametophytes and sporophytes of four algae

**Figure 4:**
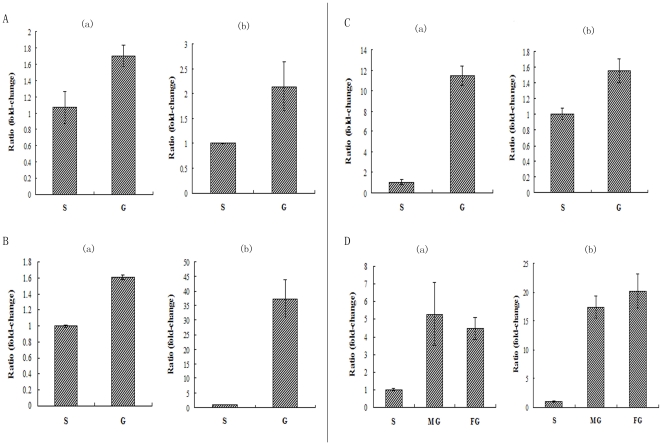
Real-time PCR analysis for the relative expression level (fold change) of rbcL gene in gametophyte and sporophyte of *P. yezoensis*, *P. haitanensis*, *B. fuscpurpurea* and *L. japonica*. The cDNA template was amplified and detected by SYBR green using primers of rbcL gene and two reference genes for each alga. A, *P. yezoensis*. B, *P. haitanensis*. C, *B. fuscopurpurea*. D, *L. japonica*. A-(a), B-(a), C-(a) and D-(a) are the results that use *18S* as the reference gene. A-(b) and B-(b) are the results that use *GAPDH* as the reference gene. C-(b) and D-(b) are the results that use *ACTIN* as the calibratorreference gene. S, Sporophyte. G, Gametophyte. MG, Male gametophyte. FG, Female gametophyte. Data are the mean value of three independent experiments (±SD).

**Figure 5:**
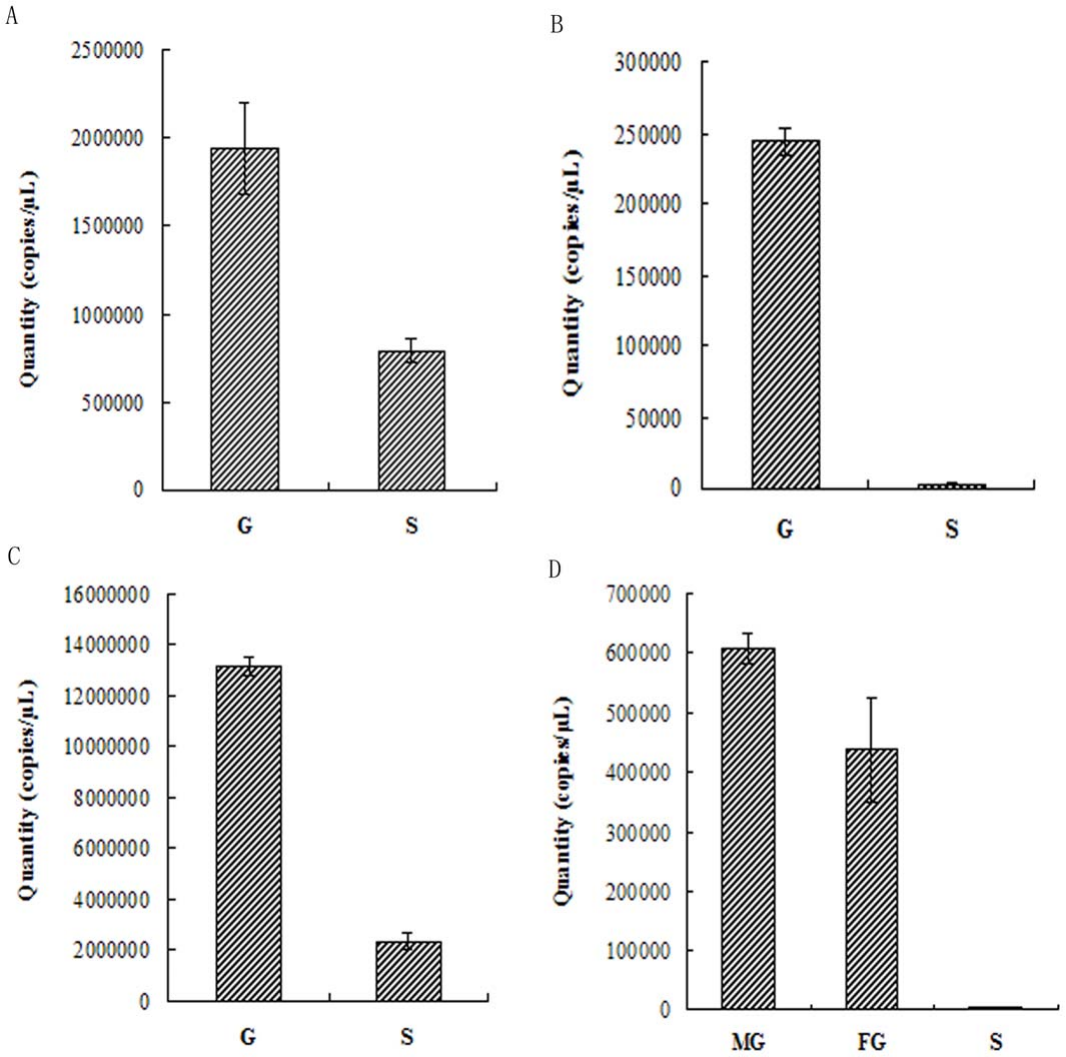
Real-time PCR analysis for the absolute quantification (copy number, copies/µL) of rbcL gene in gametophyte and sporophyte of *P. yezoensis*, *P. haitanensis*, *B. fusc-purpurea* and *L. japonica*. Serial diluted cDNAs of each standard samples and unknown samples were amplified and detected by SYBR green and primers of rbcL gene. The quantity (copy number, copies/µL) of rbcL gene of each sample was calculated according to the corresponding standard curve, which was generated by plotting the Ct value against the logarithm of the quantities of the standard samples. A, *P. yezoensis*. B, *P. haitanensis*. C, *B. fuscopurpurea*. D, *L. japonica.* S, Sporophyte. G, Gametophyte. MG, Male gametophyte. FG, Female gametophyte. Data are the mean value of three independent experiments (±SD).

Relative and absolute quantification of qPCR were carried out to determine differences in the relative (Fig. 4) and absolute expression level of the rbcL gene (Fig. 5) respectively between the gametophytes and sporophytes of *P. yezoensis*, *P. haitanensis*, *B. fuscopurpurea* and *L. japonica*. The amplification specificity for the genes of 18S, rbcL, GAPDH and ACTIN were determined by analyzing the dissociation curves of PCR products. Only one peak presented in the dissociation curves for both genes, indicating that the amplifications were specific. From the figures it is obvious that, in the four algae, higher levels of *rbcL* expression were observed in gametophytes with a significant difference both on relative (Fig. 4) and on absolute (Fig. 5) quantification (*P<0.01*), especially for absolute quantification, in which the expression level of *rbcL* in the gametophytes were above ten times more than that in the sporophyte (*P<0.01*) (Fig. 5).

### SDS-PAGE assay of the LSU of Rubisco

**Figure 6:**
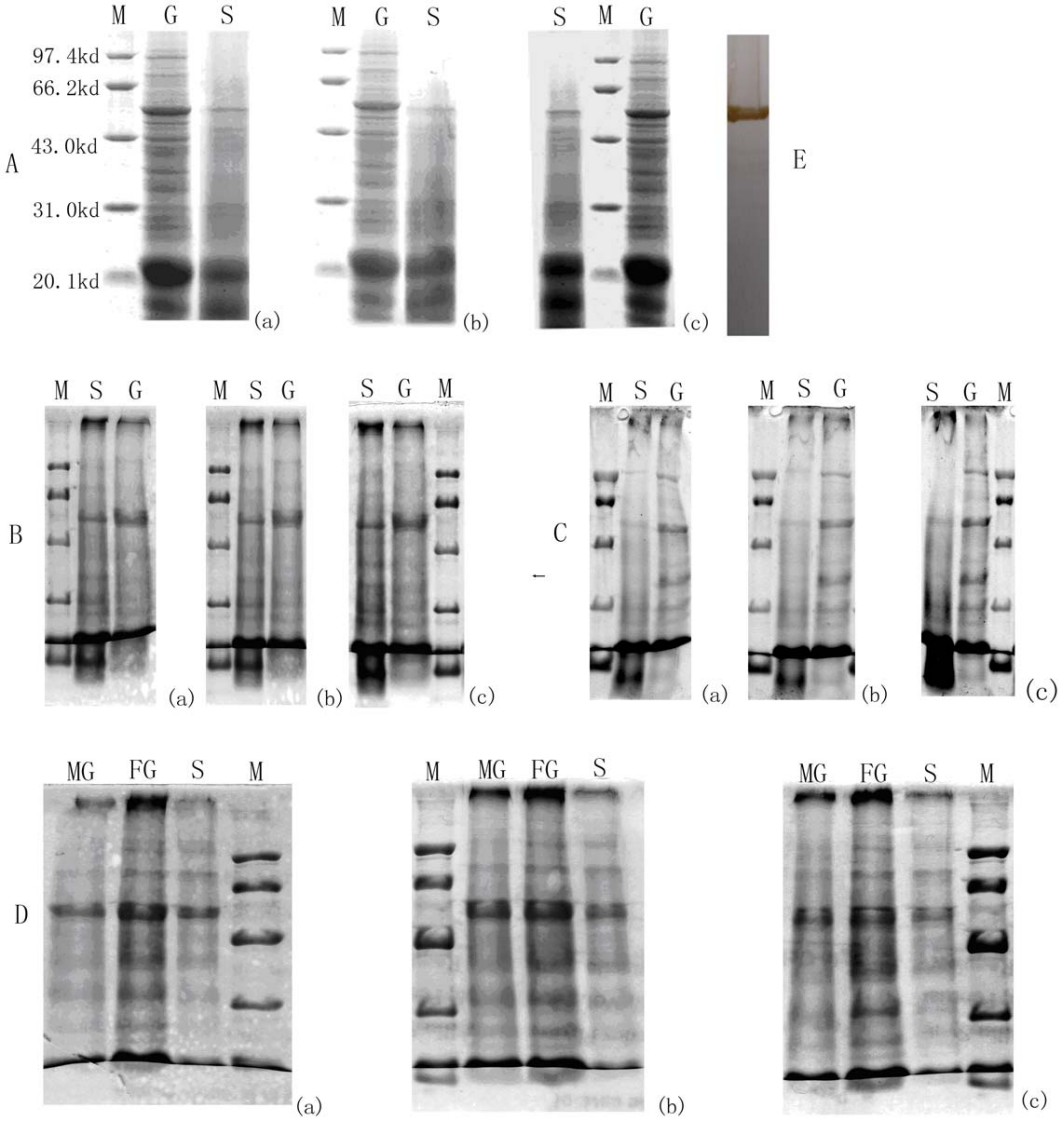
SDS-PAGE electrophoresis of the total soluble protein from gametophytes and sporophyte of *P. yezoensis*, *P. haitanensis*, *B. fusc-purpurea* and *L. japonica*. A, *P. yezoensis*. B, *P. haitanensis*. C, *B. fusc-purpurea*. D, *L. japonica*. (a), equal content of chlorophyll a per lane. (b), equal total soluble protein per lane. (c), equal fresh weight of material per lane. E, western blot of Rubisco of *P. yezoensis*. M, Maker. S, Sporophyte. G, Gametophyte. MG, Male gametophyte. FG, Female gametophyte.

The total protein of *P. yezoensis* was separated by means of SDS-PAGE, and a 55 kDa distinct band showed up in western blot analysis using Rubisco LSU antibody (Fig. 6-E). Accordingly, the 55 kDa band was identified as the LSU of Rubisco. Since the molecular weights of LSU of Rubisco from *P. yezoensis*, *P. haitanensis*, *B. fuscopurpurea* and *L. japonica* have been reported to be equal, the total protein of *P. yezoensis* was used as the control in this study, to mark the position of Rubisco LSU.

The content of LSU of Rubisco in the algal gametophytes and sporophytes was compared, using SDS-PAGE. In all of the four species (including gametophytes and sporophytes) involved in this study, a protein band at 55 kDa was detected, which represents the LSU of Rubisco (Fig. 6), and similar results were obtained in the four algal species. Loading volumes were determined according to the equal weight of chlorophyll a (Fig. 6-a), the equal weight of total protein (Fig. 6-b), or the equal fresh weight of living materials (Fig. 6-c). The LSU of Rubisco was expressed much more abundantly in the gametophytes than in the sporophytes (Fig. 6).

### Initial carboxylase activity assay of Rubisco

The initial carboxylase activity of Rubisco from gametophytes and sporophytes of *P. yezoensis*, *P. haitanensis*, *B. fuscopurpurea* and *L. japonica* was determined according to the equal fresh weight of living material, the equal weight of total protein, and the equal weight of chlorophyll a, respectively and the detail results were shown in Table 3. From Table 3 it can be seen that, the Rubisco activity rate was about 9-fold greater (*P<0.01*) in the gametophyte than in the sporophyte in *P. yezoensis* — on the basis of equal weight of chlorophyll a and equal fresh weight of living materials. For *P. haitanensis*, the activity of Rubisco was approximately 2.5 times greater (*P<0.01*) in the gametophyte than in the sporophyte on the basis of equal weight of chlorophyll a, equal fresh weight, and equal weight of total protein. As for *B. fuscopurpurea* and *L. japonica*, the ratio of Rubisco activity in the gametophyte and sporophyte reached more than 10 times (*P<0.01*) on the basis of equal weight of chlorophyll a in *B. fuscopurpurea* and equal weight of fresh living material and total protein in *L. japonica*. All the results indicated that the initial carboxylase activities of Rubisco from the gametophytes were much higher than those from the sporophytes.

**Table 3:** Initial carboxylase activity of Rubisco from different generations of *P. yezoensis*, *P. haitanensis*, *L. japonica* and *B. fuscopurpurea*.

Algal materials	Initial carboxylase activity of Rubisco		
	mmol CO_2_/mg·chla·min	mmol CO_2_/mg·pro·min	mmol CO_2_/g·F_w_·min
*L. japonica* S	3.4×10^−3^±1.30E-4	1.2×10^−4^±7.90E-06	4.8×10^−4^±1.20E-05
*L. japonica* M	4.1×10^−3^±5.50E-5	1.2×10^−3^±1.90E-04	5.3×10^−3^±3.60E-05
*L. japonica* F	4.0×10^−3^±1.80E-4	4.1×10^−3^±6.10E-05	5.3×10^−3^±1.40E-04
*P. haitanensis* S	1.0×10^−1^±8.00E-03	2.4×10^−2^±2.00E-03	1.4×10^−1^±2.20E-02
*P. haitanensis* G	2.7×10^−1^±5.10E-02	5.9×10^−2^±6.00E-03	3.6×10^−1^±3.00E-02
*B. fuscopurpurea* S	1.7×10^−2^±9.50E-04	5.2×10^−3^±1.20E-04	2.1×10^−2^±1.40E-03
*B. fuscopurpurea* G	5.7×10^−1^±1.30E-02	3.3×10^−2^±3.00E-03	2.1×10^−1^±6.00E-03
*P. yezoensis* S	2.8×10^−3^±1.30E-04	2.3×10^−4^±1.10E-05	3.8×10^−3^±1.60E-04
*P. yezoensis* G	4.5×10^−2^±2.50E-03	9.4×10^−4^±2.20E-05	4.3×10^−2^±1.90E-03

Data are the mean value of three independent experiments (±SD).

S : sporophyte;

G : gametophyte;

M : male gametophyte;

F : female gametophyte.

### Assay of photosynthetic rate

**Figure 7:**
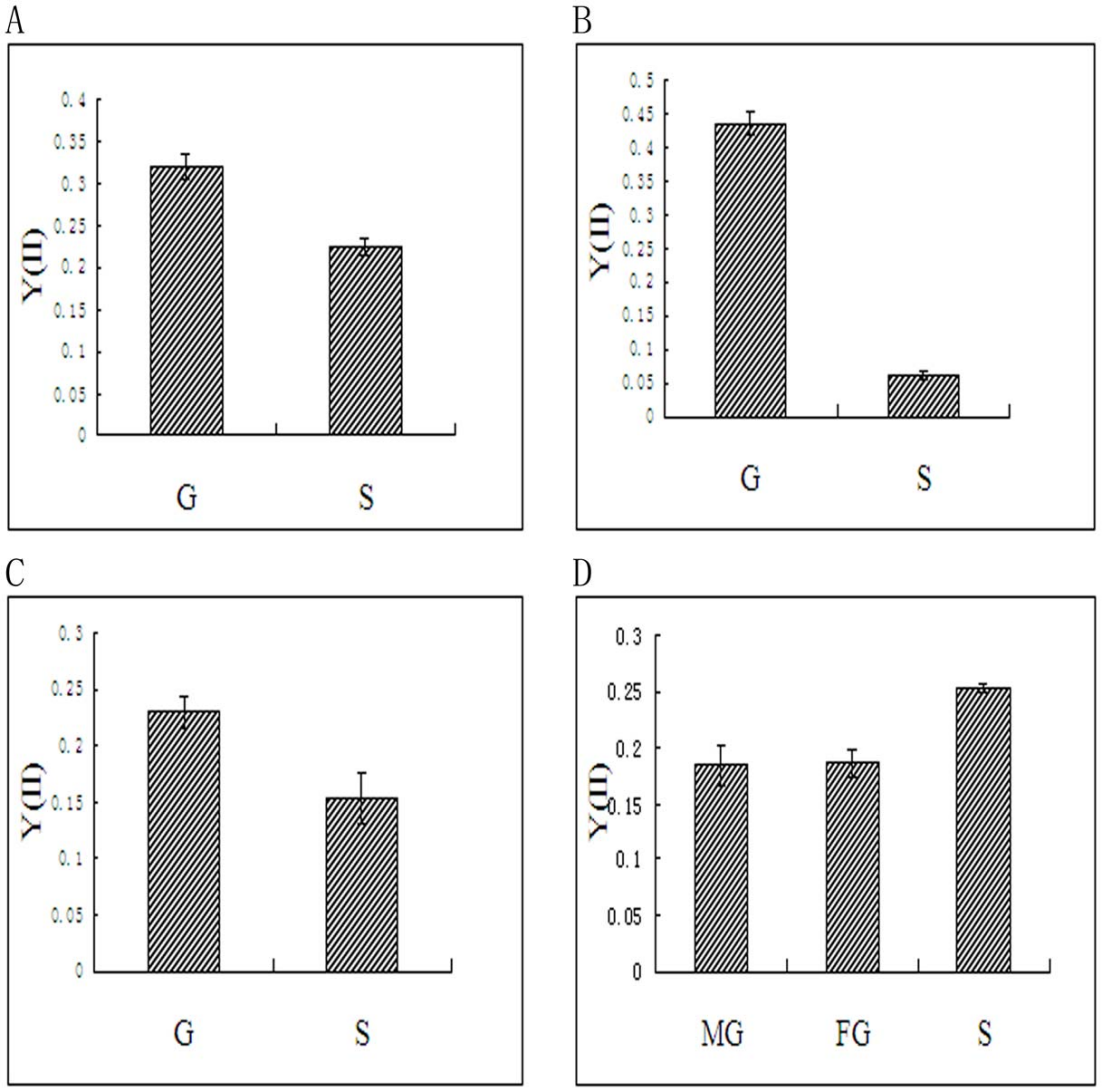
Comparison of the effective PS II quantum yield (Y II) between gametophyte and sporophytes of *P. yezoensis*, *P. haitanensis*, *B. fuscopurpurea* and *L. japonica*. A, *P. yezoensis*. B, *P. haitanensis*. C, *B. fuscopurpurea*. D, *L. japonica*. S, Sporophyte. G, Gametophyte. MG, Male gametophyte. FG, Female gametophyte. Data are the mean value of three independent experiments (±SD).

**Figure 8:**
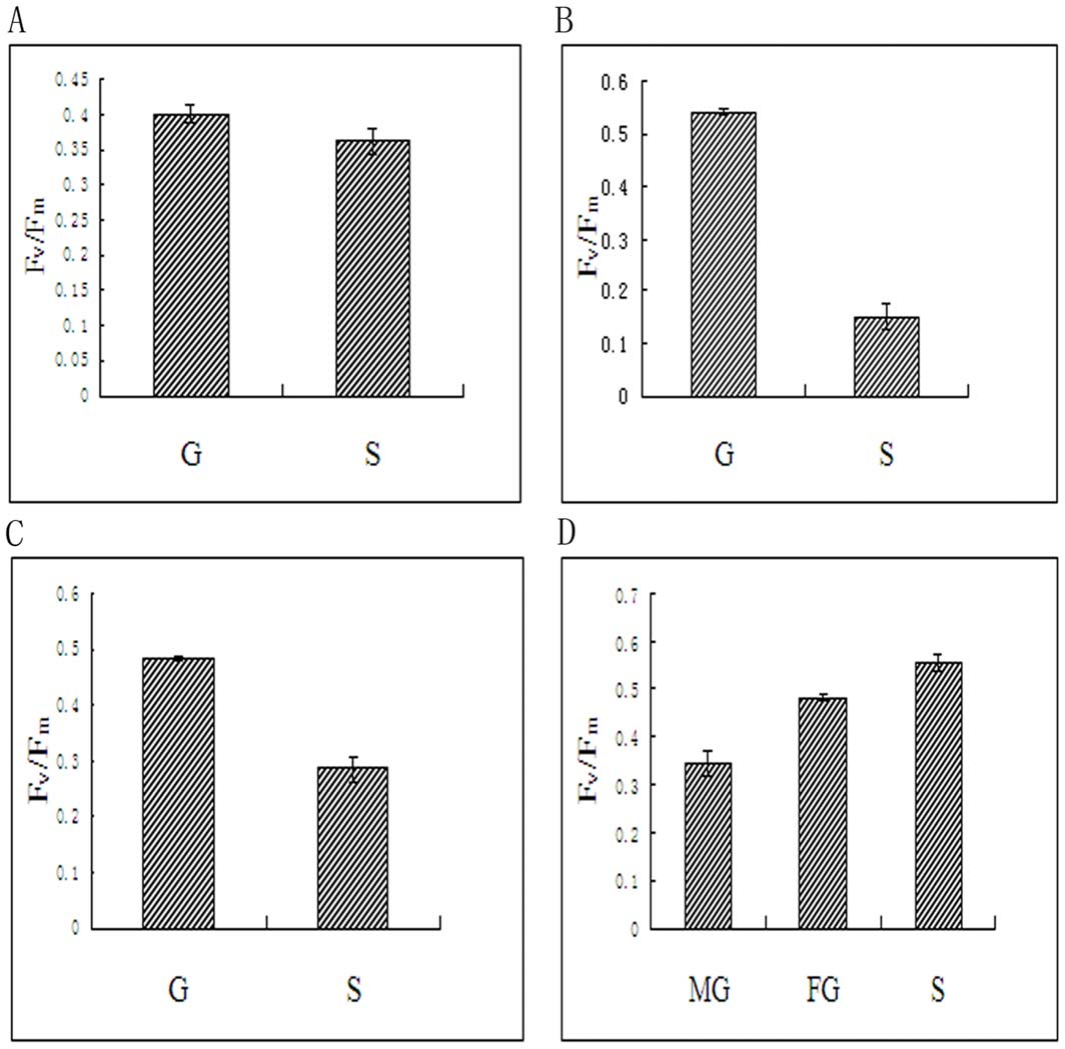
Comparison of the optimum quantum yield (F_v_/F_m_) between gametophytes and sporophytes of *P. yezoensis*, *P. haitanensis*, *B. fuscopurpurea* and *L. japonica*. A, *P. yezoensis*. B, *B. fuscopurpurea*. C, *L. japonica*.A, *P. yezoensis*. B, *P. haitanensis*. C, *B. fuscopurpurea*. D, *L. japonica*. S, Sporophyte. G, Gametophyte. MG, Male gametophyte. FG, Female gametophyte. Data are the mean value of three independent experiments (±SD).

The Imaging-PAM analysis was used to determine differences in the photosynthetic rate between the gametophytes and sporophytes of *P. yezoensis*, *P. haitanensis*, *B. fuscopurpurea* and *L. japonica* (Fig. 7 and 8). It was shown in the figures that, for the aglae of *P. yezoensis*, *P. haitanensis* and *B. fuscopurpurea*, the value of effective PS II quantum yield (Y II) of gametophytes was much higher than that of sporophytes (*P<0.01*) (Fig. 7). At the same time, the value of optimum quantum yield (F_v_/F_m_) of the three algae was also higher in gametophytes than in sporophytes (*P<0.01*) (Fig. 8). On the contrary, for the alga of *L. japonica*, both the value of Y II and F_v_/F_m_ were higher in sporophyte than in gametophyte (*P<0.01*) (Fig. 7, Fig. 8).

## Discussion

Generally, three standards are most frequently used in quantifying content and activity of Rubisco in seaweeds. These are: i) per unit of fresh weight of the living material, such as in *Fucus vesiculosus*, *Fucus serratus*, *Cladophora sericea*, *Ulva lactuca*, *Furcellaria fastigiata*, *Chondrus crispus*, *Cerarniurn rubrurn* [20], and *Mastocarpus stellatus* [21] with the shortage of the large deviation caused by the containing water; ii) per unit of content of chlorophyll a, such as in *Enteromorpha clathrat* [22], *Fucus vesiculosus*, *Fucus serratus*, *Cladophora sericea*, *Ulva lactuca*, *Furcellaria fastigiata*, *Chondrus crispus*, *Cerarniurn rubrurn* [20], *Udotea flabellum*, and *Codium decorficatum* [23], with the shortage of the deviation caused by the existence of a large amount of non-chlorophyll photosynthetic pigments; iii) per unit of weight of the total soluble protein, such as in *Ulva lactuca* [24], with the shortage of the deviation caused by the protein in the non-photosynthetic cells.

In order to improve accuracy, in this study the above three standards were all used in quantifying and comparing the carboxylase activity and content of Rubisco from different generations of the four seaweed species — *P. yezoensis*, *P. haitanensis*, *B. fuscopurpurea* (Rhodophyte) and *L. japonica* (Phaeophyceae) — which respectively represent the three main types of life history. Whatever standards we used, the results were highly consistent with each other. In spite of the quantification basis, both the initial carboxylase activity (Tab. 3) and the abundance of Rubisco (Fig. 6) were indeed much higher in the algal haploid gametophytes than in the diploid sporophytes. Moreover, results when using northern blot assay for *P. yezoensis* (Fig. 2) indicated that the mRNA content in the gametophyte was more abundant than that in the sporophyte. When qPCR was used to determine the relative expression level of rbcL gene in gametophytes and sporophytes of the four algae, the results showed that the level of *rbcL* expression in gametophytes was much higher than that in sporophytes (*P<0.01*) (Fig. 4, Fig. 5). All these results indicated that, in terms of levels of transcription and translation, the expression level of Rubisco was much higher in the haploid gametophyte than in the diploid sporophyte.

Although the relationships between protein activity and ploidy are still not certain, the gene dosage effect has been reported in a lot of higher plants: i.e., with the chromosome ploidy increasing, the gene copies and protein content increase accordingly. In higher plants, the relationship between protein activity and ploidy has been studied since 1960s [25]–[28]. Nevertheless, the gene dosage effect is displayed in two completely different ways in higher plants. In one case this effect is conspicuous, as indicated by such as Meyers *et al*. (1982) who demonstrated the gene dosage effect in *Medicago sativa* [29]. In the same year, Dean *et al*. (1982) reported that in *Triticum* species, the content of Rubisco was positively related to chromosome ploidy [30]. Leech *et al*. (1985) also found that the gene dosage effect exists in the *Triticum* species, but he pointed out that this did not apply in two genotypes of hexaploid *Triticum*, which may be due to effect of different genotypes [31]. Warner *et al*. (1989) found that the carboxylase activity of Rubisco also had a positive relationship with chromosome ploidy in the C4 plant *Atriplex confertifolia* [32]. In a different case, however, Bhaskaran *et al*. (1983) found that in a pollen-derived haploid *Saintpaulia ionantha*, Rubisco activity was much higher than that in other multiploids, and in 1987, they again successfully induced a haploid, *Nicotiana tabacum* cv. Burley Ky 14, from the pollen and found that, as with the haploids derived from the same pollen, the specific activity of the Rubisco to bind the CO_2_ was much higher than in the diploid parents. They speculated this might be attributed to the mono-genotype [33], [34].

According to the results of our study, in which we used four species of algae that had different dominant phases in their lifecycles, there were indications that Rubisco activity (Tab. 3) and content (Fig. 6) were higher in haploid than in diploid stages, which seemed contrary to the gene dosage effect. Results from our study were therefore quite different from the former case of gene dosage effect that existed in most of the higher plants but very similar to the latter one. We therefore speculate that the latter case could be relevant to the distinct encoding character of Rubisco in Rhodophyta and Phaeophyta. In green algae and higher plants, the LSU and SSU of Rubisco are encoded by the chloroplast genome and the nuclear genome respectively [3], and the assemblage and activity of Rubisco are regulated according to the content of the nuclear-genome-encoded SSU [35], [36]. The nucleic gene dosage effect is therefore obvious. In Rhodophyta and Phaeophyta, however, both LSU and SSU of Rubisco are encoded by an operon in the chloroplast genome [4], so nucleic gene dosage effect may only have a minimal effect on the assemblage of Rubisco.

In addition, it was reported in previous studies that gene candidates with differential expression in the sporophytes and gametophytes of some *Porphyra* species have been identified, which significantly contributed to the study of the transition mechanism in different algal generations [37], [38]. Nevertheless, as a result of the different habitats of sporophytes and gametophytes, the gene candidates contained a lot of genes that might have been transcribed as an adaptation to the environment. In C3 higher plants, the up-regulated expression of Rubisco was always associated with high rates of photosynthesis, particularly concerning primary production. And the photosynthetic rate assays (Fig. 7, Fig. 8) showed that, for the alage of *P. yezoensis*, *P. haitanensis* and *B. fuscpurpurea*, the comparison results of photosynthetic rates were corresponding to the comparison results of Rubisco contents (Fig. 6) and activities (Tab. 3) between gametophyte and sporophytes. While for *L. japonica*, the higher Rubisco content (Fig. 6) and activity (Tab. 3) in gametophyte was corresponding to the lower photosynthetic rate (Fig. 7, Fig. 8). Xu *et al*. (1991) had reported that PEPCK (Phosphoenolpyruvate carboxykinase), another important enzyme in carbon fixation in C4 plants, had very high activity in the sporophyte of *L. japonica* [39]. Reiskind *et al*. (1991) also reported that C4-like photosynthetic characteristics in the green alga *Udotea flabellum*, for which the high PEPCK activity with low PEPC (phosphoenolpyruvate carboxylase) activity was a novel characteristic [23]. Fan *et al*. (2007) found a similar result in *P. haitanensis* and speculated that a C4-like carbon fixation pathway may exist in the sporophyte of *P. haitanensis* [37]. Consequently, we assumed that in the sporophyte of these algae, the major carbon fixation pathway may be a C4-like carbon fixation pathway. Since the C4-like pathway could accumulate CO_2_ much more efficiently than would be the case for a ‘Rubisco pathway’, a high abundance of Rubisco would not be necessary. Rubisco could therefore have lower abundance and activity in the sporophytes of these algae.

Comparisons between gametophyte and sporophyte generations, on the level of mRNA content, in higher plants have been carried out since the 1980s. In angiosperms, it is reported that pollen mRNAs of different classes (i.e. the haploid gametophyte generation) were much more abundant than in the corresponding classes of mRNAs in shoots (i.e. the diploid sporophyte generation) of *Tradescantia1*sp. and *Zea mays* [40], [41]. This phenomenon was assumed to have resulted from a need to accumulate and store mRNAs prior to rapid translation during pollen germination [42]. Indeed, in most plants the germination and pollen tube growth are relatively rapid events [43], which need corresponding transcripts to be stored and translated during gametophyte germination [42]. Therefore, according to our results, we speculated that the haploid gametophyte of the four algae in our study had a higher abundance of mRNA than that in the diploid sporophyte, the purpose being to facilitate rapid accumulation and storage of transcripts for later differentiation in male and female gametophytes.

To sum up, according to our results, the expression of Rubisco was higher — in terms of the level of transcription, translation and enzymic activity — in haploid gametophytes than in diploid sporophytes. It was speculated that, for the four algae species studied, the nucleic gene dosage had little effect because of the distinct characteristics of Rubisco structure in Rhodophyta and Phaeophyta. Besides, a C4-like carbon fixation pathway may exist in the sporophytes of these algae, so a high abundance of Rubisco would not be necessary for the sporophytes. And the mRNA content in gametophytes was higher than that of sporophytes, which would serve the purpose of accumulating and storing transcripts for later male and female differentiation. The above speculations may explain the reason why Rubisco activity and content in the gametophytes were higher than that in the sporophytes.

## References

[1] Zhang G, Wang W, Zou Q (2004). Molecular biology of RuBisCO activase.. Plant Physiol Commun.

[2] Wang WG (1985). Experimental handbook of plant physiology.. Shanghai scientific & Technical Publishers.

[3] Valentin K, Zetsche K (1990). Structure of the Rubisco operon from the unicellular red alga *Cyanidium caldarium*: Evidence for a polyphyletic origin of the plastids.. Mol Gen Genet.

[4] Ga YC, Hwan SY, Han GC, Kazuhiro K, Sung MB (2001). Phylogeny of family *Sctyosiphonaceae* (Phaeophyta) from Korea based on sequences of plastid-encoded Rubisco spacer region.. Algae.

[5] Satoko I, Atsuko M, Seishiro A, Motomi I, Yasuro K (2009). Molecular adaptation of *rbcL* in the heterophyllous aquatic plant *Potamogeton*.. PlosOne.

[6] Wanner LA, Gruissem W (1991). Expression dynamics of the tomato rbcS gene family during development.. Plant Cell.

[7] Maayan I, Shaya F, Ratne K, Mani Y, Lavee S (2008). Photosynthetic activity during olive (Olea europaea) leaf development correlates with plastid biogenesis and Rubisco levels.. Physiol Plantrum.

[8] Patel M, Berry JO (2008). Rubisco gene expression in C4 plants.. J Exp Bot.

[9] Cabello-Pasini A, S Alberte R (2001). Enzymatic regulation of photosynthetic and light-independent carbon fixation in *Laminaria setchellii* (Phaeophyta), *Ulva lactuca* (Chlorophyta) and *Iridaea cordata* (Rhodophyta).. Revista Chilena de Historia Natural.

[10] Schwender J, Goffman F, Ohlrogge JB, Shachar-Hill Y (2004). Rubisco without the Calvin cycle improves the carbon efficiency of developing green seeds.. Nature.

[11] Zhang BY, Yang F, Wang GC, Peng G (2009). Cloning and quantitative analysis of the carbonic anhydrase gene from Porphyra yezoensis.. J Phycol.

[12] Schmittgen TD, Zakrajsek BA, Mills AG, Corn V, Singer MJ (2000). Quantitative reverse transcription-ploymerase chain reaction to study mRNA decay: comparison of endpoint and real-time methods.. Analytical Biochemistry.

[13] Bustin SA (2000). Absolute quantification of mRNA using real-time reverse transcription polymerase chain reaction assays.. Journal of Molecular Endocrinology.

[14] Bradford MM (1976). A rapid and sensitive method for the quantitation of microgram quantities of protein utilizing of protein dye dinding.. Anal Biochem.

[15] Jeffrey SW, Humphrey GF (1975). New spectrophotometric equations for determining chlorophylls a, b, c_1_ and c_2_ in higher plants, algae and natural phytoplankton. *Biochem.*. Physiol Pflanzen.

[16] Laemmli UK (1970). Cleavage of structural proteins during the assembly of the head of bacteriophage T4.. Nature.

[17] Malcolm MJ, Nguyen NY, Liu TY (1988). Reproducible high yield sequencing of proteins electrophoretically separated and transferred to an inert support.. The Journal of Biological Chemistry.

[18] Gerard VA, Driscoll T (1996). A spectrophotometric assay for RuBisCO activity: application to the kelp *Laminaria saccharina* and implications for radiometric assays.. J Phycol.

[19] Lin AP, Wang GC, Yang F, Pan GH (2009). Photosynthetic parameters of sexually different parts of *Porphyra katadai* var. *hemiphylla* (Bangiales, Rhodophyta) during dehydration and re-hydration.. Planta.

[20] Beer S, Sand-Jensen K, Vindbaek MT, Nielsen SL (1991). The carboxylase activity of RuBisCO and the photosynthetic performance in aquatic plants.. Oecologia.

[21] Dudgeon SR, Davison IR, Vadas RL (1989). Effect of freezing on photosynthesis of intertidal macroalgae: relative tolerance of Chondrus crispus and Mastocarpus stellatus (Rhodophyta).. Mar Biol.

[22] He PM, Wu QL, Wu WN, Lu W, Zhang DB (2004). Pyrenoid ultrastructure and molecular localization of RuBisCO and RuBisCO activase in *Enteromorpha clathrata*.. J fish China.

[23] Reiskind JB, Bowes G (1991). The role of phosphoenolpyruvate carboxykinase in a marine macroalga with C4-like photosynthetic characteristics.. Proc Natl Acad Sci USA.

[24] Bischof K, Kräbs G, Wiencke C, Hanelt D (2002). Solar ultraviolet radiation affects the activity of ribulose-1, 5-bisphosphate carboxylase-oxygenaseand the composition of photosynthetic and xanthophyll cycle pigments in the intertidal green alga *Ulva lactuca* L.. Planta.

[25] Ciferri O, Sora S, Tiboni O (1969). Effect of gene dosage on tryptophansynthetase activity in *Saccharomyces cerevisiae.*. Genetics.

[26] Carlson PS (1972). Locating genetic loci with aneuploids.. Mol Gen Genet.

[27] Demaggio AE, Lambrukos J (1974). Polyploidy and gene dosage effects on peroxidase activity in ferns. Biochem.. Genet.

[28] Guern M, Gherve L (1980). Polyploidy and aspartate transcarbamylase activity in *Hippocrepis comosa*.. Planta.

[29] Meyers SP, Nichols SL, Giani RB, Molin WT, Schrader LE (1982). Ploidy effects in isogenic populations of Alfalfa. 1. Ribulose-1, 5-bisphosphate carboxylase, soluble protein, chlorophyll and DNA of leaves.. Plant Physiol.

[30] Dean C, Leech RM (1982). Genome expression during normal leaf development. 2. Direct correlation between ribulose bisphosphate carboxylase content and nuclear ploidy in a polyploid series of wheat.. Plant Physiol.

[31] Leech RM, Leese BM, Jellings AJ (1985). Variation in cellular ribulose-l, 5-bisphosphate carboxylase content in leaves of Triticum genotypes at three levels of ploidy.. Planta.

[32] Warner DA, Edwards GE (1989). Effects of Polyploidy on Photosynthetic Rates, Photosynthetic Enzymes, Contents of DNA, Chlorophyll, and Sizes and Numbers of Photosynthetic Cells in the C_4_ Dicot *Atriplex confertifolia*.. Plant Physiol.

[33] Bhaskaran S, Smith RH, Finer JJ (1983). Ribulose Bisphosphate Carboxylase Activity in Anther-Derived Plants of Saintpaulia ionantha Wendl. Shag.. Plant Physiol.

[34] Bhaskaran S, Burdick PJ, Smith RH (1987). Crystallization of Ribulose, 1, 5-Bisphosphate Carboxylase of High Specific Activity from Anther-Derived Haploid Plants of *Nicotiana tabacum.*. J Exp. Bot.

[35] Rodermel S (1999). Subunit control of RuBisCO biosynthesis-a relic of an endosymbiotic past.. Photosvn Res.

[36] Miao YG, Li LR (1996). Molecular hybridization of RuBisCO subunits between rice and tobacoo.. Acta Phytophysiol Sin.

[37] Fan XL, Fang YJ, Hu SN, Wang GC (2007). Generation and analysis of 5318 express sequence tags (ESTs) from filamentous sporophyte of *Porphyra haitanensis* (Rhodophyte).. J Phycol.

[38] Asamizu E, Nakajima M, Kitade Y, Saga N, Nakamura Y (2003). Comparison of RNA expression profiles between the two generations of *Porphyra yezoensis* (Rhodophyta), based on expressed sequence tag frequency analysis.. J Phycol.

[39] Xu ZM, Yao NY, Li JZ (1991). Studies on the activity of PEPck in *L. japonica*.. Mar Sci.

[40] Willing RP, Mascarenhas JP (1984). Analysis of the complexity and diversity of mRNA from pollen and shoots of *Tradescantia*.. Plant Physiol.

[41] Willing RP, Bashe D, Mascarenhas JP (1988). An analysis of the quantity and diversity of messenger RNAs from pollen and shoots of *Zea mays*.. Theor Appl Genet.

[42] Twell D, Scott RJ, Stead AD (1994). The diversity and regulation of gene expression in the pathway of male gametophyte development..

[43] Mascarenhas JP (1989). The male gametophyte of flowering plants.. The Plant Cell.

